# Direct molecular versus culture-based assessment of Gram-positive cocci in biopsies of patients with major abscesses and diabetic foot infections

**DOI:** 10.1007/s10096-015-2428-4

**Published:** 2015-07-05

**Authors:** M. H. T. Stappers, F. Hagen, P. Reimnitz, J. W. Mouton, J. F. Meis, I. C. Gyssens

**Affiliations:** Department of Internal Medicine, Radboud University Medical Center, P.O. Box 9101, 6500 HB Nijmegen, The Netherlands; Department of Medical Microbiology and Infectious Diseases, Canisius-Wilhelmina Hospital, Nijmegen, The Netherlands; Hasselt University, Hasselt, Belgium; Bayer Healthcare Pharmaceuticals, Wuppertal, Germany; Department of Medical Microbiology, Radboud University Medical Center, Nijmegen, The Netherlands; Department of Medical Microbiology and Infectious Diseases, Erasmus MC, Rotterdam, The Netherlands

## Abstract

Major abscesses and diabetic foot infections (DFIs) are predominant subtypes of complicated skin and skin structure infections (cSSSIs), and are mainly caused by *Staphylococcus aureus* and β-hemolytic streptococci. This study evaluates the potential benefit of direct pathogen-specific real-time polymerase chain reaction (PCR) assays in the identification of causative organisms of cSSSIs. One-hundred and fifty major abscess and 128 DFI biopsy samples were collected and microbial DNA was extracted by using the Universal Microbe Detection kit for tissue samples. Pathogen-specific PCRs were developed for *S. aureus* and its virulence factor Panton–Valentine leukocidin (PVL), *Streptococcus pyogenes*, *S. agalactiae*, *S. dysgalactiae*, and the *S. anginosus* group. Identification by pathogen-specific PCRs was compared to routine culture and both methods were considered as the gold standard for determination of the sensitivity and specificity of each assay. Direct real-time PCR assays of biopsy samples resulted in a 34 % higher detection of *S. aureus*, 37 % higher detection of *S. pyogenes*, 18 % higher detection of *S. agalactiae*, 4 % higher detection of *S. dysgalactiae* subspecies *equisimilis*, and 7 % higher detection of the *S. anginosus* group, compared to routine bacterial culture. The presence of PVL was mainly confined to *S. aureus* isolated from major abscess but not DFI biopsy samples. In conclusion, our pathogen-specific real-time PCR assays had a higher yield than culture methods and could be an additional method for the detection of relevant causative pathogens in biopsies.

## Introduction

Major abscesses and diabetic foot infections (DFIs) are the predominant subtypes of a spectrum of infections termed complicated skin and skin structure infections (cSSSIs). The Food and Drug Administration (FDA) defined cSSSIs as infections of the deeper soft tissues, involving surgical intervention or a significant underlying disease state that complicates the response to treatment. Superficial infections located in an anatomical site in which the chance of involvement of anaerobic or Gram-negative pathogens is high should also be considered as cSSSIs [1, 2]. cSSSIs are associated with significant morbidity and mortality, as well as prolonged and expensive hospitalizations [3]. The management of cSSSIs involves surgical debridement of the infection, combined with antibiotic therapy [4].

Gram-positive cocci, in particular *Staphylococcus aureus* and β-hemolytic streptococci, are the leading causative organisms of cSSSIs [2, 4]. In a recent multicenter randomized clinical trial, 65 % of the cultured isolates consisted of Gram-positive cocci (*S. aureus* 33 % and β-hemolytic streptococci 15 %), whereas Gram-negative bacilli (28 %) and anaerobes (7 %) were found to a lesser extent [5], but geographical differences exist in the type and amount of species isolated [6]. Correct and rapid identification of pathogens is crucial for clinical decision-making and optimal antibiotic therapy. Up to now, routine bacteriological assessment of biopsies from cSSSIs relies upon culture, which, in order to be successful, requires viable pathogens in tissue and the use of suitable culture conditions for growth. Difficult to culture pathogens, those present in low numbers or that died before/during sampling of the infected tissue make detection by culture complicated and time-consuming. This may result in low sensitivity and underestimated bacterial prevalence. Several molecular assays, such as pathogen-specific, broad-range, and multiplex polymerase chain reaction (PCR) assays, either directly on clinical samples or cultured isolates, have been developed in recent years to improve bacteriological detection [7, 8]. This study evaluates the potential benefit of direct, pathogen-specific real-time PCR assays on clinical samples in the identification of the causative organisms of cSSSIs.

Biopsy samples of 150 major abscesses and 128 DFIs were collected during a multicenter clinical trial involving patients with cSSSIs [5]. Detection of the pathogens *S. aureus* and *Streptococcus* species by real-time PCR directly on DNA isolated from these clinical cSSSI samples was compared to routine cultures.

## Materials and methods

### Definitions

cSSSIs were characterized as infections of bacterial origin that required hospitalization, initial parenteral therapy for ≥48 h, and which met at least one of the following criteria: deep soft tissue involvement; significant surgical intervention, including drainage and/or debridement; and association with an underlying comorbid condition. Major abscesses were defined as collections of pus associated with extensive cellulitis, requiring surgical intervention followed by antibiotic therapy. DFIs were characterized as infections occurring below the ankle in patients with confirmed diabetes [5].

### Collection of major abscess and DFI biopsy samples

From a population of 813 cSSSI patients included in a large randomized, multicenter clinical trial [5], performed from September 2006 to June 2008, 389 patients gave informed consent to participate in this substudy. Of these, the first visit, prestudy treatment biopsy samples were selected, resulting in the inclusion of 150 major abscess and 128 DFI samples from 225 cSSSI patients. Samples were collected via biopsy of tissue or bone, curettage of the wound, or aspiration of purulent discharge. After collection, samples were directly stored in preservation medium (BBL Port-A-Cul Transport vial, Becton Dickinson, Franklin Lakes, NJ, USA) and transported within 72 h to the central laboratory (Eurofins Medinet SAS, Plaisir, France) [5]. On arrival, samples were split into two, one part for immediate culture and identification by using standard clinical laboratory procedures, and the other part was stored at −80 °C for subsequent DNA isolation and PCR analysis.

### Bacterial DNA extraction from biopsy samples

Pathogen DNA was manually extracted by using the MolYsis Universal Microbial Detection kit for tissue samples (Molzym, Bremen, Germany), following the manufacturer’s instructions. Briefly, selective lysis of human cells is performed, followed by degradation of human DNA. After washing the pellet, a second round of DNA extraction is performed to release DNA from bacterial and fungal cells. This approach enables an increased sensitivity and specificity for pathogen DNA, since interfering non-target human DNA is no longer present [9]. Pathogen DNA extraction was carried out in a laminar flow cabinet to prevent contamination. Prior to further handling, the DNA samples were stored at −20 °C.

### Real-time PCR primer and probe design

To detect *S. agalactiae*, in both subspecies of *S. dysgalactiae* and *S. pyogenes*, the *recA* gene was chosen as the target, based on the results from previous studies [10, 11]. Partial *recA* sequences of clinically relevant *Streptococcus* species were extracted from GenBank (accession numbers EU156792–EU156872), an alignment was generated in MEGA v6 [12], and primer and probe sets were developed to make sure that there was no overlap with other *Streptococcus* species. The specificity of the primers and probes were in silico tested by a BLAST search in GenBank.

A previously described multiplex real-time PCR assay was used to detect the *S. aureus*-specific fragment Sa442 and the Panton–Valentine leukocidin (PVL) gene [13].

The duplex real-time PCRs were performed in reaction volumes of 20 μL consisting of 10 μL 2× LC480 Probe Master Mix (Roche Diagnostics), 0.1 μL of each primer and 0.04 μL of both probes (100pmol/μL; Eurogentec, Brussels, Belgium), 1.72 μL ddH_2_O, and 8 μL sample DNA. The PCRs were performed with the following settings: initial denaturation step for 10 min at 95 °C, 50 cycles of 1 s at 95 °C, 12 s at 60 °C, followed by measuring the fluorescence signal, and a cooldown step for 30s at 40 °C.

For detection of members of the *S. anginosus* group (*S. anginosus*, *S. constellatus*, and *S. intermedius*, also termed the *S. milleri* group), a set of primers was developed based on an alignment made in MEGA v6 of the *recN* sequences EU917226–EU917315 [12, 14]. The PCRs were performed in 10 μL reaction volumes, containing 5 μL 2× LC480 Probe Master Mix (Roche Diagnostics, Almere, the Netherlands), 0.5 μL of each primer (10pmol/μL; Eurogentec), 0.5 μL SYTO82 (40 μM; Molecular Probes, Eugene, OR, USA), 2.5 μL ddH_2_O, and 1 μL sample DNA. The following PCR program was used: initial denaturation for 10 min at 95 °C, 35 cycles of 5 s at 95 °C, 5 s at 60 °C, and 10 s at 72 °C, followed by a melting curve analysis at 65 °C to 95 °C. Fluorescence was measured after each extension step.

The primer and probe sequences of all assays are provided in Table 1.

**Table 1:** Primer and probe sequences

Specificity	Forward primer	Probe	Reverse primer
S. aureus*	5’-ACGACTARATAAACGCTCATTCG-3’	5’-HEX-TGAAATCTCATTACGTTGCATCGGA-BHQ1-3’	5’-GACGGCTTTTACATACAGAACACA-3’
PVL*	5’-AAAAAGGCTCAGGAGATACAAGTG -3’	5’-Cy5-TGGCAGAAATATGGATGTTACTCATGC-BHQ2-3’	5’-TGCCATAGTGTGTTGTTCTTCTAGT-3’
*S. pyogenes*	5’-TTGGAAACGATAGCTAATACCG-3’	5’-HEX-CGCATGTTAGTAATTTAAAAGGGGCA-BHQ1-3’	5’-CGCAGGTCCATCTCATAGTG-3’
*S. agalactiae*		5’-FAM-TGTTAGTTATTTAAAAGGAGCAATTGC-BHQ1-3’	
*S. dysgalactiae equisimilis*		5’-HEX-CCCATGTTAAACATTTAAAAGGTGCA-BHQ1-3’	
*S. dysgalactiae dysgalactiae*		5’-FAM-AATGGAGGACCCATGTCTTTCATTT-BHQ1-3’	
*S. anginosus* group	5’-TGGACAGCATTTGGTGGATA-3’	n/a	5’-GCTTACGCAACTGACGATACTG-3’

*This assay was previously published by Hopman et al. [13]

### Real-time PCR assays and analysis

The analytic specificity was tested by applying the newly developed *Streptococcus* assays on a set of 62 clinically relevant *Streptococcus* reference strains. The sensitivity of the *Streptococcus* assays was performed by applying two-step dilution series of the relevant type strains, starting with an input ranging from 0.01 ng to 4.9 fg per reaction. The equivalent of genomic copies per reaction was calculated based on the genome sizes for each of the *Streptococcus* species [15, 16].

### Statistical analysis

McNemar’s test was performed to study differences between real-time PCR and culture-based assessment of pathogenic presence, for major abscesses and DFIs separately. The results for major abscesses and DFIs were combined for the determination of the sensitivity, specificity, positive predictive value (PPV), and negative predictive value (NPV). Both culture and real-time PCR were considered as the gold standard.

## Results

### Validation of species-specific *Streptococcus* real-time PCR assays and analysis

The *S. agalactiae* real-time PCR assay was found to be positive for the included strains; however, atypical amplification curves were observed for two *S. anginosus* strains. The *S. pyogenes* assay, included in the duplex real-time PCR with *S. agalactiae*, was found to be exclusively positive for strains that belong to this species. The *S. dysgalactiae* duplex real-time PCR assay was positive for the included subspecies *dysgalactiae* and *equisimilis*. However, the assay for subspecies *equisimilis* showed an atypical amplification curve for one strain of *S. constellatus* and *S. cristatus*.

The lower limit of detection was found to be at the 9th dilution step for the targets *S. agalactiae*, both subspecies of *S. dysgalactiae* and *S. pyogenes*, equivalent of ~39 fg DNA per reaction, equal to ~17 genomic copies per reaction. For the intercalating dye-based assay for *S. anginosus*, *S. constellatus* and *S. intermedius* assay, the lower limit of detection was found to be at the 8th dilution step, equal to 78 fg or ~37 genomic copies per reaction.

### Real-time PCR versus culture-based assessment of *S. aureus* prevalence in major abscesses and DFIs

The first visit biopsy samples of 150 major abscesses and 128 DFIs were collected, and the identification of *S. aureus* was performed by real-time PCR and culture. Of the 150 major abscess samples, 81 % were positive for *S. aureus* by real-time PCR, whereas 44 % were culture-positive for *S. aureus*. None of the culture-positive samples were real-time PCR-negative (Table 2). Of the 128 DFI samples, 88 % were positive for *S. aureus* by real-time PCR, whereas 57 % were culture-positive for *S. aureus*. One culture-positive sample was found to be real-time PCR-negative (Table 2). Subsequent real-time PCR determination of the cultured strain from the culture-positive, real-time PCR-negative biopsy sample confirmed the presence of *S. aureus*. Statistical analysis using McNemar’s test showed a significant difference between *S. aureus* real-time PCR and culture-based assessment for major abscesses (*p* < 0.001) and DFIs (*p* < 0.001). The sensitivity, specificity, PPV, and NPV for the *S. aureus* real-time PCR and culture are shown in Table 3.

**Table 2:** Real-time polymerase chain reaction (real-time PCR) versus culture-based assessment of Gram-positive coccal prevalence in major abscesses and diabetic foot infections

	Major abscesses				Diabetic foot infections		
	Real-time PCR-positive	Real-time PCR-negative	Total		Real-time PCR-positive	Real-time PCR-negative	Total
*S. aureus*							
Culture-positive	66	0	66	Culture-positive	72	1	73
Culture-negative	55	29	84	Culture-negative	41	14	55
Total	121	29	150	Total	113	15	128
*S. pyogenes*							
Culture-positive	23	0	23	Culture-positive	1	0	1
Culture-negative	104	23	127	Culture-negative	18	109	127
Total	127	23	150	Total	19	109	128
*S. agalactiae*							
Culture-positive	5	3	8	Culture-positive	18	10	28
Culture-negative	42	100	142	Culture-negative	21	79	100
Total	47	103	150	Total	39	89	128
*S. dysgalactiae* subspecies *equisimilis*							
Culture-positive	3	1	4	Culture-positive	16	1	17
Culture-negative	0	146	146	Culture-negative	12	99	111
Total	3	147	150	Total	28	100	128
*S. anginosus* group							
Culture-positive	16	7	23	Culture-positive	6	1	7
Culture-negative	13	114	127	Culture-negative	13	108	121
Total	29	121	150	Total	19	109	128

**Table 3:** Sensitivity, specificity, positive predictive value (PPV), and negative predictive value (NPV) of real-time polymerase chain reaction (real-time PCR) and culture-based assessment of Gram-positive coccal prevalence in major abscesses and diabetic foot infections

Species	Reference	Assay	Sensitivity (%)	Specificity (%)	PPV (%)	NPV (%)
*S. aureus*	Culture	Real-time PCR	99	31	59	98
	Real-time PCR	Culture	59	98	99	31
*S. pyogenes*	Culture	Real-time PCR	100	86	16	100
	Real-time PCR	Culture	16	100	100	86
*S. agalactiae*	Culture	Real-time PCR	64	74	27	93
	Real-time PCR	Culture	27	93	64	74
*S. dysgalactiae equisimilis*	Culture	Real-time PCR	91	95	61	99
	Real-time PCR	Culture	61	99	91	95
*S. anginosus* group	Culture	Real-time PCR	73	90	46	97
	Real-time PCR	Culture	46	97	73	90

### Real-time PCR-based assessment of PVL versus *S. aureus* prevalence in major abscesses and DFIs

The presence of *S. aureus* virulence factor PVL was determined by real-time PCR and correlated to *S. aureus* real-time PCR-positive biopsies. Of interest, the majority of *S. aureus* real-time PCR-positive major abscess samples were also PVL real-time PCR-positive (89 %). In contrast, a minority of *S. aureus* real-time PCR-positive DFI samples were PVL real-time PCR-positive (14 %). In both groups of major abscesses and DFIs, two samples were PVL real-time PCR-positive but not *S. aureus* real-time PCR-positive (Table 4).

**Table 4:** Real-time polymerase chain reaction (real-time PCR)-based assessment of Panton–Valentine leukocidin (PVL) versus *Staphylococcus aureus* prevalence in major abscesses and diabetic foot infections

	Major abscesses				Diabetic foot infections		
	*S. aureus* real-time PCR-positive	*S. aureus* real-time PCR-negative	Total		*S. aureus* real-time PCR-positive	*S. aureus *real-time PCR-negative	Total
PVL real-time PCR-positive	108	2	110	PVL real-time PCR-positive	16	2	18
PVL real-time PCR-negative	13	27	40	PVL real-time PCR-negative	97	13	110
Total	121	29	150	Total	113	15	128

### Real-time PCR versus culture-based assessment of *S. pyogenes* prevalence in major abscesses and DFIs

Of the 150 major abscess samples, 85 % were positive for *S. pyogenes* by real-time PCR, whereas 15 % were culture-positive for *S. pyogenes*. None of the culture-positive samples were real-time PCR-negative (Table 2). Of the 128 DFI samples, 15 % were positive for *S. pyogenes* by real-time PCR, whereas 1 % were culture-positive for *S. pyogenes*. The only culture-positive sample was also real-time PCR-positive (Table 2). Statistical analysis showed a significant difference between *S. pyogenes* real-time PCR and culture-based assessment for major abscesses (p < 0.001) and DFIs (*p* < 0.001). The sensitivity, specificity, PPV, and NPV for the *S. pyogenes* real-time PCR and culture are shown in Table 3.

### Real-time PCR versus culture-based assessment of *S. agalactiae* prevalence in major abscesses and DFIs

Of the 150 major abscess samples, 31 % were positive for *S. agalactiae* by real-time PCR, whereas 5 % were culture-positive for *S. agalactiae*. Three culture-positive samples were real-time PCR-negative (Table 2). Of the 128 DFI samples, 30 % were positive for *S. agalactiae* by real-time PCR, whereas 22 % were culture-positive for *S. agalactiae*. Ten culture-positive samples were real-time PCR-negative (Table 2). Subsequent real-time PCR determination of the cultured strains from the 13 culture-positive, real-time PCR-negative biopsy samples confirmed the presence of *S. agalactiae* of four strains. The other nine strains were negative for *S. agalactiae* real-time PCR. Statistical analysis showed a significant difference between *S. agalactiae* real-time PCR and culture-based assessment for major abscesses (*p* < 0.001) but not DFIs (*p* > 0.05). The sensitivity, specificity, PPV, and NPV for the *S. agalactiae* real-time PCR and culture are shown in Table 3.

### Real-time PCR versus culture-based assessment of *S. dysgalactiae* prevalence in major abscesses and DFIs

Of the 150 major abscess samples, 2 % were positive for *S. dysgalactiae* subspecies *equisimilis* by real-time PCR, whereas 3 % were culture-positive for *S. dysgalactiae* subspecies *equisimilis*. One of the culture-positive samples was real-time PCR-negative (Table 2). Of the 128 DFI samples, 22 % were positive for *S. dysgalactiae* subspecies *equisimilis* by real-time PCR, whereas 13 % were culture-positive for *S. dysgalactiae* subspecies *equisimilis*. One DFI sample was found to be culture-positive but real-time PCR-negative (Table 2). Subsequent real-time PCR determination of the cultured strains from the two culture-positive, real-time PCR-negative biopsy samples confirmed the presence of *S. dysgalactiae* subspecies *equisimilis* of one strain. The other strain was negative for *S. dysgalactiae* subspecies *equisimilis* real-time PCR. Statistical analysis showed a significant difference between *S. dysgalactiae* subspecies *equisimilis* real-time PCR and culture-based assessment for DFIs (*p* < 0.01) but not major abscesses (*p* > 0.05). The sensitivity, specificity, PPV, and NPV for the *S. dysgalactiae* subspecies *equisimilis* real-time PCR and culture are shown in Table 3.

None of the 150 major abscess samples were real-time PCR or culture positive for *S. dysgalactiae* subspecies *dysgalactiae*. Of the 128 DFI samples, only one sample was positive for *S. dysgalactiae* subspecies *dysgalactiae* by real-time PCR but culture-negative, and, also, one sample was culture-positive for *S. dysgalactiae* subspecies *dysgalactiae* but real-time PCR-negative. Subsequent real-time PCR determination of the cultured strain from the culture-positive, real-time PCR-negative biopsy sample was negative for *S. dysgalactiae* subspecies *dysgalactiae* but positive for *S. dysgalactiae* subspecies *equisimilis*.

### Real-time PCR versus culture-based assessment of *S. anginosus* group prevalence in major abscesses and DFIs

*S. anginosus*, *S. constellatus*, and *S. intermedius* together constitute the *S. anginosus* group. Of the 150 major abscess samples, 19 % were positive for members of the *S. anginosus* group by real-time PCR, whereas 15 % were culture-positive for members of the *S. anginosus* group. Seven culture-positive samples were real-time PCR-negative (Table 2). Of the 128 DFI samples, 15 % were positive for members of the *S. anginosus* group by real-time PCR, whereas 6 % were culture-positive for members of the *S. anginosus* group. One culture-positive sample was real-time PCR-negative (Table 2). Subsequent real-time PCR determination of the cultured strains from the eight culture-positive, real-time PCR-negative biopsy samples confirmed identification of the *S. anginosus* group of six strains. Of the two other strains, one was negative for *S. anginosus* group real-time PCR and the other strain was not viable. Statistical analysis showed a significant difference between *S. anginosus* group real-time PCR and culture-based assessment for DFIs (*p* < 0.01) but not major abscesses (*p* > 0.05). The sensitivity, specificity, PPV, and NPV for the *S. anginosus* group real-time PCR and culture are shown in Table 3.

## Discussion

This study is the first to demonstrate that the use of direct real-time PCR versus culture-based assessment for the determination of pathogens in clinical biopsy samples of patients with major abscesses and DFIs resulted in an increased detection of all studied cSSSI pathogens: *S. aureus*, *S. pyogenes*, *S. agalactiae*, *S. dysgalactiae*, and *S. anginosus* group.

The current routine practice for the detection and identification of bacterial pathogens in cSSSI is culture of biopsy samples collected from the site of infection. Development of direct real-time PCR assays on bacterial DNA isolated from biopsy samples resulted in a higher detection of 34 % for *S. aureus*, 37 % for *S. pyogenes*, 18 % for *S. agalactiae*, 4 % for *S. dysgalactiae* subspecies *equisimilis*, and 7 % for the *S. anginosus* group compared to standard cultures. No differences were observed for *S. dysgalactiae* subspecies *dysgalactiae*, which could be due to its low prevalence in human infections [17]. Significant differences were found between real-time PCR and culture assessment for *S. aureus* (major abscesses and DFIs), *S. pyogenes* (major abscesses and DFIs), *S. agalactiae* (major abscesses), *S. dysgalactiae* subspecies *equisimilis* (DFIs), and *S. anginosus* group (DFIs). Possible explanations for the real-time PCR-positive, culture-negative biopsy samples are: culture is less sensitive than real-time PCR for the detection of organisms, real-time PCR detects nonviable organisms in contrast to culture, splitting of the biopsy sample into two parts for culture and real-time PCR, contamination of the real-time PCR reagents, cross-reactivity of the real-time PCR assay with DNA from other organisms or human origin not controlled for during the development of the assay inducing false-positives.

In contrast, our real-time PCR assays did not identify some pathogens that were grown in culture. Possible explanations for the culture-positive, real-time PCR-negative biopsy samples are: real-time PCR is less sensitive than culture for the detection of organisms, incorrect determination during culture, low prevalence of bacterial DNA in the biopsy sample, splitting of the biopsy sample into two parts for culture and real-time PCR, low DNA yield or poor quality DNA after extraction or the presence of factors that inhibit the real-time PCR assay, poor target specificity, and/or competition between primers and probes within the developed real-time PCR not controlled for during the development of the assay.

Other studies evaluating the potential of direct real-time PCR in the detection of the organisms studied here frequently found comparable or increased detection rates compared to culture methods. The detection of (methicillin-resistant) *S. aureus* by real-time PCR was similar to culture in samples from skin and soft tissue infections and osteoarticular infections [18, 19], and increased in a screening for MRSA colonization [20]. In addition, the detection of *S. pyogenes* by real-time PCR was similar to culture in throat swabs from suspected pharyngitis [21, 22]. Furthermore, similar and increased detection rates of *S. agalactiae* were found in screening samples of vaginal and neonatal colonization [23–25], and increased detection was observed by real-time PCR for *S. agalactiae* in cerebrospinal fluid and blood from patients suspected of meningitis and sepsis [26].

Overall, combined detection by either culture and/or real-time PCR resulted in 85 % of samples positive for *S. aureus*, 68 % positive for *S. pyogenes*, 36 % positive for *S. agalactiae*, 12 % positive for *S. dysgalactiae* subspecies *equisimilis*, 0.7 % positive for *S. dysgalactiae* subspecies *dysgalactiae*, and 20 % positive for *S. anginosus* group. This again underlines the importance of Gram-positive cocci in cSSSIs. Interestingly, there are differences in the species present in major abscesses and DFIs. *S. pyogenes* was mainly detected in biopsies from major abscesses, whereas *S. dysgalactiae* subspecies *equisimilis* was mainly found in biopsies from diabetic foot infections. Other identified pathogens were similarly present in both major abscesses and DFIs. In addition, a clear distinction was observed between major abscesses and DFIs for PVL-positive *S. aureus*. 73 % of the major abscesses, but only 14 % of the DFIs, were positive for PVL. PVL is a bi-component pore-forming toxin, encoded by the *lukS*-PV and *lukF*-PV genes, and is associated with lysis of leukocytes [27]. A recent meta-analysis confirmed the association between PVL and *S. aureus* skin and skin structure infections, which was not present for *S. aureus* invasive infections, such as pneumonia, musculoskeletal infections, and bacteremia [28].

In an attempt to evaluate the reliability of the real-time PCR and culture, we have calculated the sensitivity, specificity, PPV, and NPV. Both techniques were separately considered as the gold standard, as it is unknown which technique represents the “true” presence of pathogens in the biopsy samples.

In conclusion, current routine bacteriological assessment of biopsies by culture is time-consuming, requires viable pathogens, culture conditions suitable for growth, and results in lower detection sensitivity and may underestimate bacterial prevalence. However, given the fast and superior detection of cSSSI pathogens by real-time PCR, our study indicates that molecular analysis can be an additional method for the detection of bacteria in clinical samples.
